# Effects of Different Ecological Restoration Treatments on Plant Functional Group Characteristics and Nitrogen and Phosphorus Stoichiometry in Alpine Semi-Humid Sandy Land

**DOI:** 10.3390/plants15203083

**Published:** 2026-10-09

**Authors:** Yanling Li, Nairui Yang, Hongyu Qian, Yuhong Tong, Changjiang Song, Ranxi Gou, Li Lin, Li Zhou

**Affiliations:** 1 School of Management, Sichuan Agricultural University, Chengdu 611130, China; 2 College of Resources, Sichuan Agricultural University, Chengdu 611130, China; 3 College of Landscape Architecture, Sichuan Agricultural University, Chengdu 611130, China; 4 School of Materials Science and Engineering, Sichuan University of Science and Engineering, Zigong 643000, China; 5 Yibin Academy of Agricultural Sciences, Yibin 644600, China

**Keywords:** alpine sandy land, ecological restoration, plant functional groups, plant diversity, stoichiometric ratio

## Abstract

Ecological restoration of alpine sandy lands plays an important role in promoting vegetation recovery and enhancing ecosystem functions. However, the response characteristics of plant functional groups and nitrogen (N) and phosphorus (P) dynamics under different restoration treatments remain unclear. Taking three typical ecological restoration treatments in the alpine semi-humid sandy land on the eastern edge of the Qinghai–Tibet Plateau—fencing + sand barrier establishment + mixed grass seeding (MG), fencing + sand barrier establishment + shrub planting alone (SA), and fencing + sand barrier establishment + shrub–grass combination (SG)—as the study objects, and unrestored natural sandy land as the control (CK), this study investigated the characteristics of plant biomass and diversity, as well as the content and stoichiometric characteristics of N and P in the plant–litter system under different restoration treatments, and analyzed the response relationships between plant diversity and changes in N and P in the plant–litter system. The results showed that all three restoration treatments increased plant community biomass and diversity indices. Among them, the SG treatment had the highest aboveground biomass and diversity, whereas the MG treatment had the largest belowground biomass. All three restoration treatments increased the N and P contents of herbaceous plants and litter, with the greatest increases observed under the SG treatment. The results for plant functional groups showed that legumes had the highest N content, while forbs had the highest P content. The N:P ratios of aboveground plant parts ranged from 5.85 to 8.82 and were lower in the SA and SG treatments, suggesting that plants under the restoration treatments may be N-limited. In conclusion, the SG treatment is the most effective restoration approach for promoting vegetation recovery and nutrient content in alpine sandy lands. These results provide a scientific basis for selecting ecological restoration treatments for alpine sandy lands and for improving restoration effectiveness and efficiency.

## 1. Introduction

Alpine grasslands are widely distributed in the semi-humid region of the eastern Qinghai–Tibet Plateau. This area is also home to the world’s largest plateau peat swamp wetlands and is one of China’s key biodiversity regions, giving it an extremely important ecological and environmental status [1]. However, due to long-term natural and anthropogenic factors such as global climate change, wind erosion, rodent damage, ditch drainage, and overgrazing, the alpine grasslands in this region have experienced severe degradation, with prominent desertification in localized areas. This poses a serious threat to regional ecological security and the sustainable development of animal husbandry [2]. In recent years, to curb the further expansion of desertification, a series of ecological restoration efforts have been undertaken in the eastern Qinghai–Tibet Plateau, leading to the development of typical ecological restoration treatments primarily based on mixed grass seeding, shrub planting, and shrub–grass combinations [1]. In the process of ecological restoration, suitable sand-fixing shrubs and grasses play a vital role by preventing wind erosion, fixing sand, improving soil conditions, and enhancing the local micro-environment. These actions promote the recovery of surface vegetation, gradually leading to the formation of a stable shrub–grass ecosystem, which is crucial for the restoration of desertified lands [3,4]. The biogenic elements nitrogen (N) and phosphorus (P) are essential macronutrients for plant growth and development. Their concentrations play a key role in ecosystem evolution and stability [5,6]. Studying the characteristics of element accumulation in ecosystems under ecological restoration is of great significance for revealing the cycles and migration-transformation patterns of biogenic elements, as well as for regulating ecological restoration measures in alpine sandy lands.

As key factors in maintaining ecosystem balance, the accumulation, allocation, and stoichiometric ratios of N and P in plants and litter reflect, to some extent, the internal cycling and return mechanisms of vegetation nutrients, and are influenced by multiple factors [7]. Plant nutrient uptake rates also affect the stoichiometric characteristics of litter, subsequently influencing litter decomposition rates and nutrient release [8]. Through efficient nutrient uptake, plants produce litter with higher C:N and C:P [8]. Some studies have confirmed that litter with high C:N ratios, which is rich in difficult-to-decompose substances such as lignin, decomposes more slowly, leading to enhanced microbial immobilization of soil nitrogen and potentially exacerbating ecosystem nitrogen limitation [9,10]. Ecological stoichiometry is a discipline that studies the balance of energy and chemical elements in ecosystems [10], and is of great significance for investigating the supply–demand relationships of N and P nutrients in the plant–litter system. Plants absorb and fix N and P nutrients from the soil through their root systems, while simultaneously returning N and P to the surface in the form of litter. This indicates a coupling relationship among soil N and P supply, the return of N and P during litter decomposition, and plant N and P nutrient demand [11,12]. Therefore, studying the content and stoichiometric characteristics of N and P in plants and litter from the perspective of the plant–litter continuum is crucial for understanding the distribution of N and P between vegetation and litter in ecosystems.

Plant diversity is a crucial factor influencing ecosystem function and stability, and is closely linked to ecosystem N and P cycling processes [13]. Plant functional groups can effectively elucidate the roles of plant species with similar adaptive characteristics in ecosystems and reflect the dynamic changes of vegetation in response to environmental conditions. They serve as an important basis for evaluating changes in ecosystem structure and function and are directly associated with ecosystem N and P dynamics [14]. For instance, Li et al. [15] found that different plant species and functional groups exhibit distinct nutrient acquisition strategies and resorption efficiencies, leading to variations in their N and P stoichiometric characteristics. Xu et al. [16] also suggested that the uptake efficiencies and stoichiometry of leaf N and P in plants show different responses to treatments depending on plant functional groups, species traits, climate, and soil nutrient conditions. Ecological restoration induces changes in plant diversity and functional group biomass, which in turn regulate the retention and turnover of nutrients in plants. This affects plant N and P fixation and the transfer of N and P from plants to the soil, leading to alterations in the content of ecosystem N and P nutrients. Moreover, these changes vary among different ecological restoration treatments [14,17]. Currently, research on N and P contents in alpine regions has primarily focused on soils [18]. The response of N and P contents in herbaceous plant functional groups to ecological restoration, as well as their relationship with plant diversity, remains insufficiently supported by research.

Therefore, this study focuses on three typical ecological restoration treatments (planting mixed grasses treatment; planting *Salix cupularis* shrubs alone; planting shrub with *Salix cupularis* plus mixed grasses treatment) in alpine sandy lands to investigate the characteristics of plant biomass and diversity, as well as the content and stoichiometric characteristics of N and P in the plant–litter system under different restoration treatments on the eastern edge of the Qinghai–Tibet Plateau. The response relationships between plant diversity and changes in N and P in the plant–litter system were also analyzed. Three scientific hypotheses were formulated: (1) Different ecological restoration treatments significantly alter the composition and structure of plant functional groups, thereby affecting plant community productivity and species diversity. (2) Different ecological restoration treatments influence the content and stoichiometric characteristics of N and P in the plant–litter system by modifying plant functional group composition. (3) There is a significant coupling relationship between changes in plant diversity and the contents of N and P in the plant–litter system. The findings will contribute to understanding the biogeochemical cycling processes of N and P under ecological restoration in alpine sandy lands, while providing a scientific basis for selecting ecological restoration treatments and improving restoration effectiveness and efficiency in these regions.

## 2. Results

### 2.1. Biomass of Plant Communities and Functional Groups

The results showed that all ecological restoration treatments increased the aboveground, underground, and litter biomass of the plant communities (Table 1). The total aboveground biomass and litter biomass across the three restoration treatments followed the order SG > SA > MG, with significant differences among treatments (*p* < 0.05). The total underground biomass followed the order MG > SG > SA, with significant differences among treatments (*p* < 0.05).

**Figure 1:**
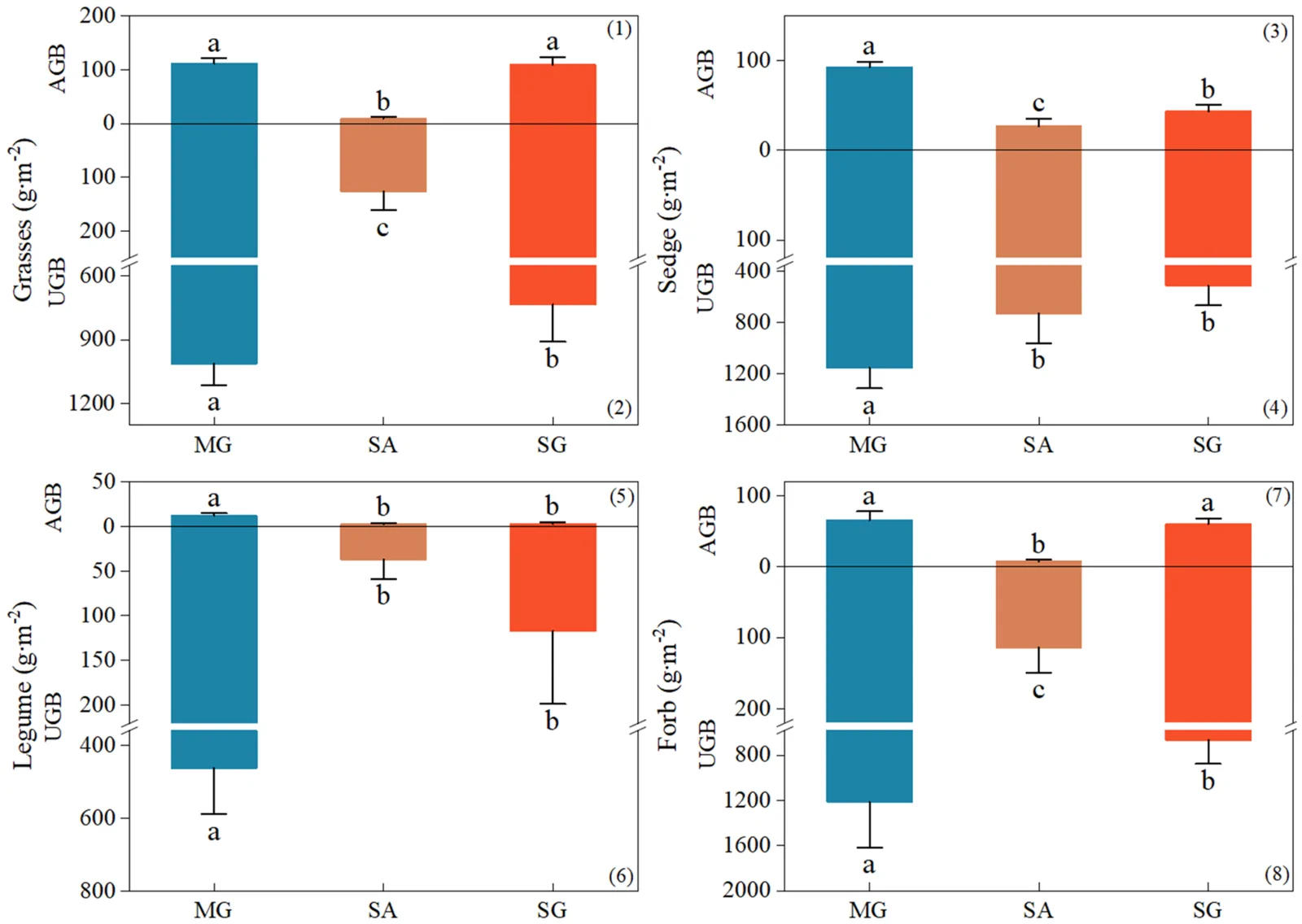
AGB (1) and UGB (2) of grasses under different ecological restoration treatments; AGB (3) and UGB (4) of sedge; AGB (5) and UGB (6) of legume; AGB (7) and UGB (8) of forb. Note: MG, planting mixed grasses treatment; SA, planting shrub with *Salix cupularis* alone treatment; SG, planting shrub with *Salix cupularis* plus mixed grasses treatment. AGB, aboveground biomass; UGB, underground biomass; Grasses, grasses functional groups; Sedge, sedge functional groups; Legume, legume functional group; Forb, forb functional groups. Data were presented as means ± SD (*n* = 4). Different lowercase letters indicated significant differences among treatments at *p* < 0.05.

As shown in Figure 1, grasses were the dominant plants in the MG and SG treatments, while sedges were the dominant plants in the SA treatment. Within the same restoration treatment, both aboveground and underground biomass of the legumes functional group were lower than those of the other functional groups. Significant differences were observed in the aboveground and underground biomass of plant functional groups among different restoration treatments. The aboveground biomass of grasses, sedges, legumes, and forbs in the MG treatment was significantly higher than that in the SA treatment (*p* < 0.05). The aboveground biomass of grasses, sedges, legumes, and forbs in the MG treatment was significantly higher than that in the SA and SG treatments (*p* < 0.05).

### 2.2. Plant Diversity

The diversity indices of the aboveground vegetation communities differed among the three restoration treatments, with no significant differences between the MG and SG treatments (*p* > 0.05) (Table 2). The trends for the Magarlef and Shannon–Wiener indices were SG > MG > SA, while those for Pielou and Simpson were SG = MG > SA.

### 2.3. N and P Contents and Stoichiometric Ratios in Salix cupularis Shrubs and Herbaceous Plant Communities

**Figure 2:**
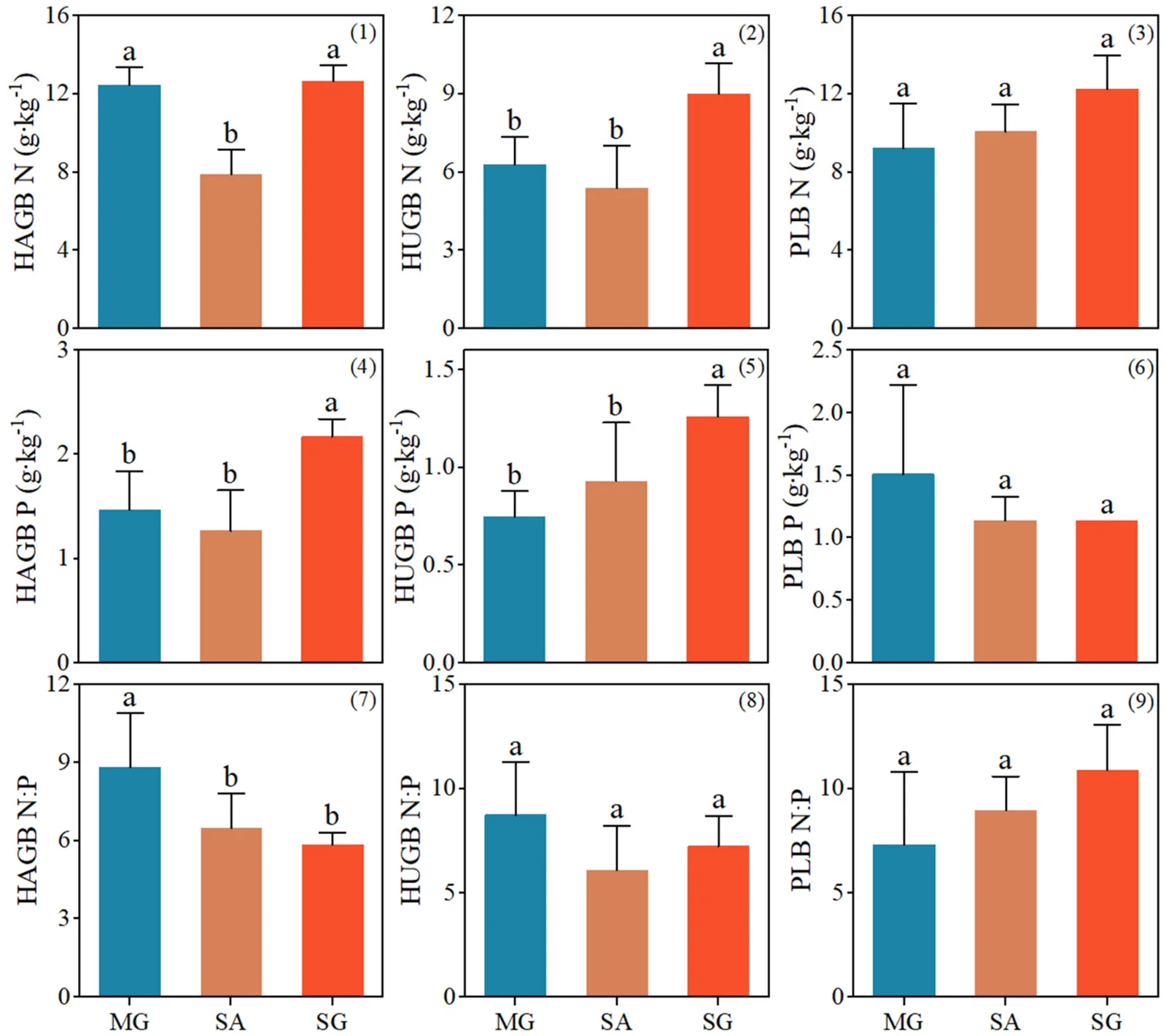
N and P contents and their stoichiometric ratios in herbaceous plants and litter under different ecological restoration treatments. Note: MG, planting mixed grasses treatment; SA, planting shrub with *Salix cupularis* alone treatment; SG, planting shrub with *Salix cupularis* plus mixed grasses treatment. HAGB, herbaceous plants aboveground; HUGB, herbaceous plants underground; PLB, plant litter biomass. Data were presented as means ± SD (*n* = 4). Different lowercase letters indicated significant differences among treatments at *p* < 0.05.

The results showed that all ecological restoration treatments increased the N content in the aboveground parts of herbaceous plants and community litter (Figure 2 (1–3)). The aboveground N content of herbaceous plants in the MG and SG treatments was significantly higher than that in the SA treatment (*p* < 0.05), and the underground N content of herbaceous plants in the SG treatment was significantly higher than that in the MG and SA treatments by 42.91% and 67.23%, respectively (*p* < 0.05). No significant differences were observed in litter N content among the three restoration treatments (*p* > 0.05).

Similar to the changes in N content, all ecological restoration treatments significantly increased the P content in the aboveground parts of herbaceous plants and community litter (Figure 2 (4–6)). The aboveground P content of herbaceous plants in the SG treatment was significantly higher than that in the MG and SA treatments by 47.37% and 71.06%, respectively (*p* < 0.05). The trend of changes in underground P content of herbaceous plants was similar to that of aboveground P content. No significant differences were found in litter P content among the three restoration treatments (*p* > 0.05).

As shown in Figure 2 (7–9), the aboveground N:P ratios of herbaceous plants ranged from 5.85 to 8.82. Compared with the MG treatment, the aboveground N:P ratios of herbaceous plants in the SA and SG treatments were significantly reduced by 26.38% and 33.66%, respectively (*p* < 0.05). However, no significant differences were observed in the underground parts of herbaceous plants or community litter among the three restoration treatments (*p* > 0.05).

**Figure 3:**
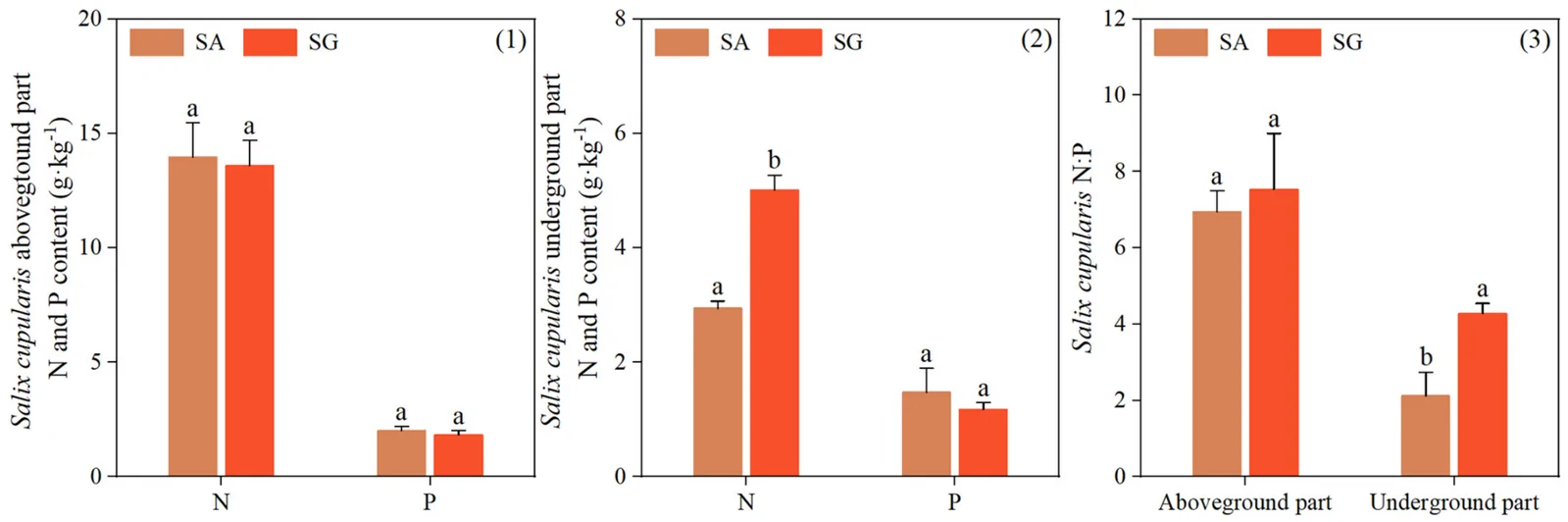
Effects of ecological restoration treatment on N and P contents and stoichiometric ratio of *Salix cupularis*. Note: SA, planting shrub with *Salix cupularis* alone treatment; SG, planting shrub with *Salix cupularis* plus mixed grasses treatment. HAGB, herbaceous plants aboveground; HUGB, herbaceous plants underground; PLB, plant litter biomass. Data were presented as means ± SD (*n* = 4). Different lowercase letters indicated significant differences among treatments at *p* < 0.05.

The results showed that compared with the SA treatment, the SG treatment significantly increased the underground N content of *Salix cupularis* shrubs by 2.06 g∙kg^−1^ and its N:P ratio by 2.16, while no significant differences were observed in other indicators between the two restoration treatments (*p* < 0.05) (Figure 3).

### 2.4. N and P Contents and Stoichiometric Ratios in Herbaceous Plant Functional Groups

**Figure 4:**
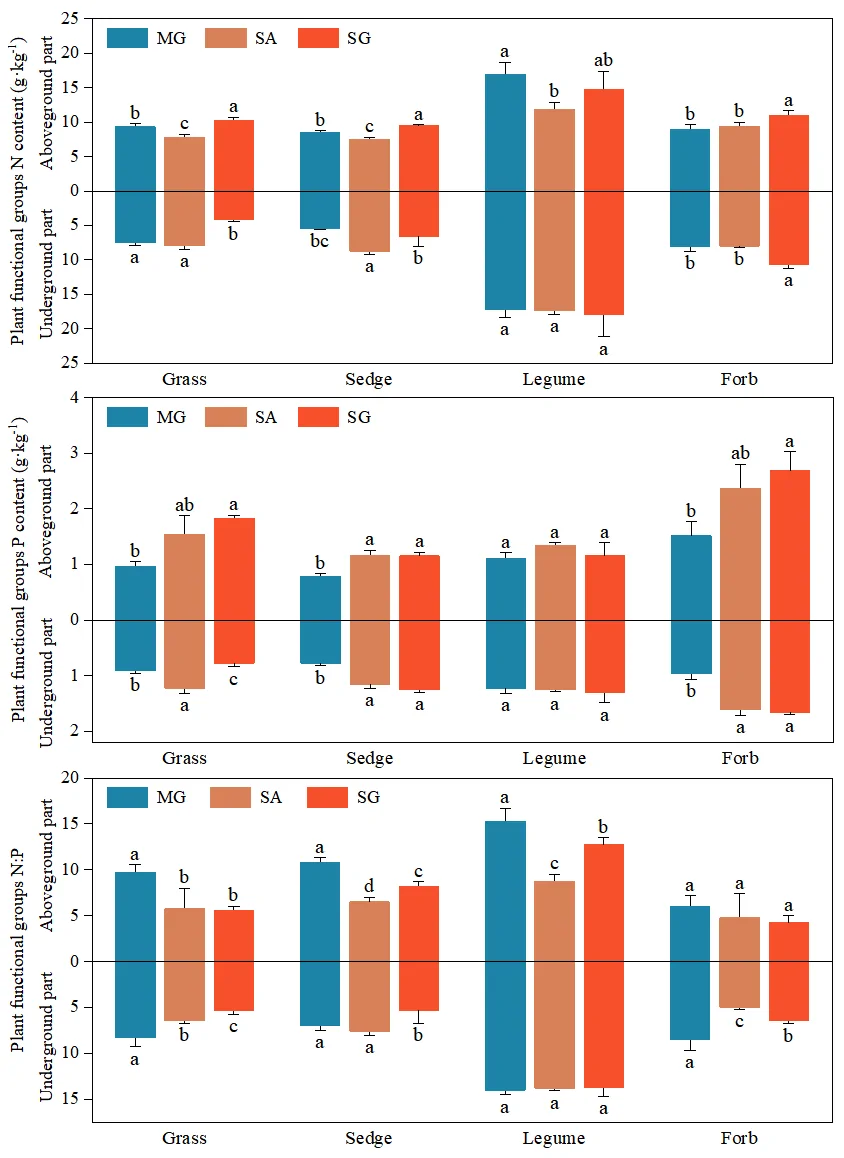
Effects of ecological restoration treatments on aboveground and underground part N and P content and stoichiometric ratio of plant functional groups. Note: MG, planting mixed grasses treatment; SA, planting shrub with *Salix cupularis* alone treatment; SG, planting shrub with *Salix cupularis* plus mixed grasses treatment. Data were presented as means ± SD (*n* = 4). Different lowercase letters indicated significant differences among treatments at *p* < 0.05.

The results showed that different restoration treatments significantly affected N contents in the above- and underground parts of various plant functional groups. Within the same restoration treatment, the aboveground and underground N contents of the legume functional group were higher than those of the other functional groups (Figure 4). For the grasses, sedge, and forb functional groups, the aboveground N content in the SG treatment was significantly higher than that in the MG and SA treatments, while for the legume functional group, the aboveground N content in the MG treatment was significantly higher than that in the SA and SG treatments (*p* < 0.05). For the grasses and sedge functional groups, the underground N content in the SA treatment was significantly higher than that in the SG treatment (*p* < 0.05). However, no significant differences were observed in the underground N content of the legume functional group among the three restoration treatments. For the forb functional group, the underground N content in the SG treatment was significantly higher than that in the MG and SA treatments (*p* < 0.05).

Different restoration treatments significantly affected the aboveground and underground P contents of various plant functional groups (*p* < 0.05), while no significant differences were found in the aboveground and underground P contents of the legume functional group among the different restoration treatments (*p* > 0.05) (Figure 4). Within the same restoration treatment, the aboveground P content of forbs was significantly higher than that of the other functional groups (*p* < 0.05). For the grasses, sedge, legume, and forb functional groups, the aboveground P content in the MG treatment was significantly lower than that in the SA and SG treatments (*p* < 0.05), while no significant difference was observed between the SA and SG treatments (*p* > 0.05). For the sedge, legume, and forb functional groups, the underground P content in the SG treatment was significantly higher than that in the MG treatment (*p* < 0.05), but showed no significant difference compared to the SA treatment (*p* > 0.05). For the grasses functional group, the underground P content in the SA treatment was significantly higher than that in the MG and SG treatments (*p* < 0.05).

Different restoration treatments significantly affected the aboveground and underground N:P ratios of various plant functional groups, while no significant differences were observed in the aboveground N:P ratio of forbs or the underground N:P ratio of legumes among the different restoration treatments (*p* > 0.05) (Figure 4). Overall, the aboveground and underground N:P ratios of the four plant functional groups in the MG treatment were significantly higher than those in the SA and SG treatments (*p* < 0.05), whereas no significant difference was found in the underground N:P ratio of the sedge functional group between the MG and SA treatments (*p* > 0.05).

### 2.5. Correlation Analysis Between Plant N and P Contents and Biomass and Species Diversity

**Figure 5:**
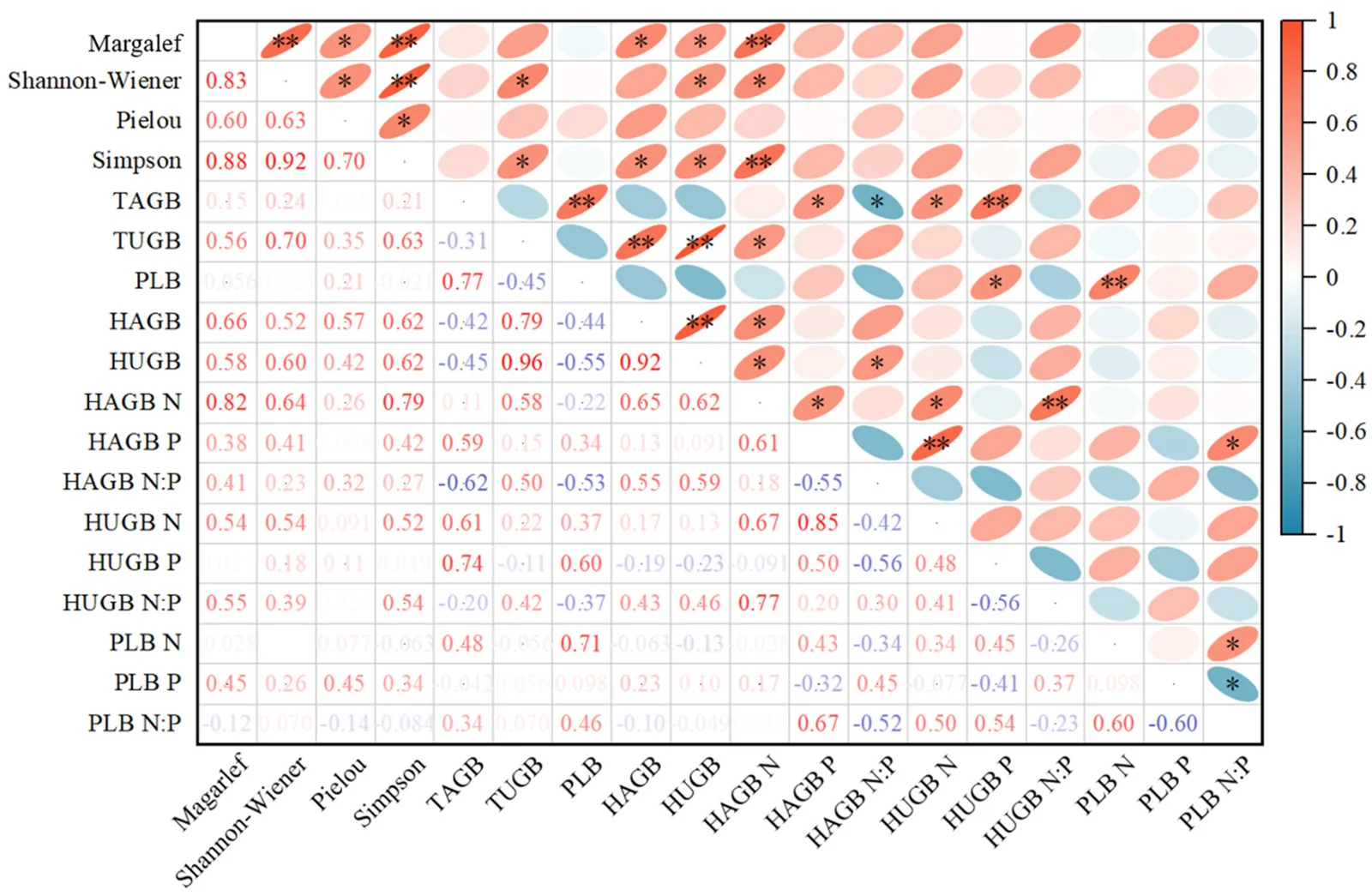
Relationship between plant biomass, diversity and N, P content. Note: TAGB, total aboveground biomass; TUGB, total underground biomass; PLB, plant litter biomass; HAGB, herbaceous aboveground biomass; HUGB, herbaceous underground biomass. * indicates that has a significant difference (*, *p* < 0.05; **, *p* < 0.01).

The correlation analysis showed that, among the plant diversity indices, the Margalef, Shannon–Wiener, and Simpson indices were all significantly positively correlated with HUGB and HAGB N (*p* < 0.05). Regarding the relationships between plant biomass and plant N and P contents, HAGB was significantly positively correlated with HAGB N, and HUGB was significantly positively correlated with HAGB N and N:P; TAGB was significantly positively correlated with HAGB P, HUGB N, and HUGB P, and significantly negatively correlated with HAGB N:P. TUGB was significantly positively correlated with HAGB N. PLB was significantly positively correlated with HUGB P and PLB P (*p* < 0.05) (Figure 5).

## 3. Discussion

### 3.1. Effects of Different Ecological Restoration Treatments on Plant Biomass and Species Diversity

During ecological restoration, the increase in plant biomass can promote soil carbon sequestration, improve soil quality, and enhance ecosystem stability [19]. The results of this study showed that all three restoration treatments increased the total aboveground and underground biomass of plant communities, which is consistent with previous research findings. Staples et al. [20], in their study on reforestation of reclaimed land in southeastern Queensland, Australia, found that ecological restoration measures increased plant aboveground biomass, which recovered to pre-reclamation levels after 30 years. Chen et al. [21], in their study on the effects of artificial and natural restoration in the Baotou light rare earth tailings pond, found that vegetation community characteristics and soil physicochemical properties were significantly improved in artificially restored areas. Yang et al. [22] studied the effects of different restoration measures in semi-arid grasslands of Inner Mongolia and found that vegetation restoration increased aboveground plant and litter biomass, and altered soil and plant N:P ratios. In this study, the aboveground biomass and litter biomass in the SG and SA treatments were significantly higher than those in the MG treatment. This is because the SG and SA treatments contain *Salix cupularis* shrubs, whose aboveground biomass is much higher than that of herbaceous plants in the MG treatment, also leading to substantial accumulation of litter. Plant biomass allocation refers to the relative biomass distribution among different plant organs. Plants allocate resources to the most needed organs, enabling them to more effectively acquire the scarcest resources and adapt to environmental changes [23]. In this study, under all three treatments, herbaceous plant biomass was primarily allocated to underground biomass. This may be because the study area was established on sandy land, where soil moisture and nutrient contents are relatively low, prompting herbaceous plants to allocate more biomass to root systems to obtain sufficient water and nutrient resources [24].

Furthermore, the pattern of plant biomass allocation depends not only on environmental factors but also, to some extent, on differences in resource acquisition strategies among plant functional types [25]. Based on major plant types and functional characteristics, meadow soil plants in alpine regions are mainly classified into four functional groups: grasses, sedges, legumes, and forbs [25,26]. Changes in plant groups reflect, to some extent, the successional trend of plant communities. An increase in the proportion of grasses and sedges is generally interpreted as indicating progressive succession of the plant community, whereas an increase in the proportion of forbs indicates community degradation [26]. In this study, plant biomass under the three ecological restoration treatments was mainly concentrated in grasses and sedges, with legumes and forbs also present, indicating that ecological restoration promotes vegetation recovery in alpine sandy lands. The higher proportion of grasses and sedges in the MG and SG treatments is consistent with—though not direct evidence of—progressive succession; this pattern could equally reflect the identity of the species that were sown and their persistence, rather than a successional transition. Dong et al. [27] suggested that perennial legumes struggle to grow and reproduce in alpine regions above 3700 m altitude, which aligns with our finding that legume biomass accounted for the smallest proportion of total community biomass.

Plant diversity is the foundation for plant communities to maintain ecosystem structure and function, and it is also an important ecological indicator reflecting the degree of ecosystem restoration [26]. Vegetation restoration in desertified grasslands of the Qinghai–Tibet Plateau has been reported to markedly increase community richness, diversity, and evenness, with the magnitude of the increase depending on the restoration measure applied [18]. Among the three restoration treatments in this study, the Magarlef, Shannon–Wiener, Pielou, and Simpson indices were highest in the SG treatment, followed by MG and SA, indicating that the shrub–grass combination showed the best restoration effect on community plant diversity [28]. The higher diversity under SG may reflect greater habitat heterogeneity, whereby the shrubs create both shaded and open microhabitats that allow shrub-associated and light-demanding herbaceous species to coexist [29]; in contrast, MG lacks the shrub layer and SA lacks a diverse herbaceous understory, so the herbaceous community under SA was dominated by a few shade-tolerant species and had the lowest richness and diversity. However, these mechanisms were not directly tested, and at least two alternative explanations cannot be excluded. First, species identity: because the sown species pool differed among treatments, part of the measured diversity is attributable to the species that were planted rather than to shrub effects per se. Second, microsite selection: because the quadrats in SA and SG were deliberately placed beneath the shrub canopy, the diversity estimates reflect the non-randomly sampled shrub understory rather than the treatment as a whole. Likewise, the significant positive correlations between diversity and biomass are consistent with—but do not demonstrate—niche complementarity among species [30]; such correlations could equally arise from a sampling effect or from the shared response of both variables to restoration intensity.

### 3.2. Effects of Different Ecological Restoration Treatments on Plant Nitrogen and Phosphorus

Ecologically, the effect of restoration is reflected at the macroscale in vegetation community recovery, and at the microscale in changes to plant nutrients [25]. N and P are core nutrients required for plant growth and development, influencing plant growth and metabolic processes [31]. In this study, all three restoration treatments exhibited high aboveground N and P contents in herbaceous plants, with the SG treatment showing the highest values overall, indicating that ecological restoration can enhance the nutrient acquisition capacity of herbaceous plants. The higher aboveground N and P contents are consistent with the expectation that more N-rich (enzymes, proteins, amino acids) and P-rich (ribosomal RNA) compounds are involved in metabolic activities [32]; however, these physiological processes were not measured and remain a hypothesis. Significant increases in underground N and P contents of herbaceous plants were observed only in the SG treatment. This pattern may be attributable to greater shading by the denser *Salix cupularis* canopy [33] and/or to a rhizosphere priming effect that activates soil N and P in the root zone [24], but neither nutrient-use efficiency nor rhizosphere priming was directly quantified here, and the higher underground values could also reflect differences in species identity between treatments rather than these mechanisms. Litter is an important component connecting soil and plants in ecosystems, and its nutrient content is significantly influenced by the dominant plant species and litter composition of the community [34]. In this study, all three restoration treatments produced litter with substantial N and P contents, which generally aligned with the N and P contents of the aboveground parts of plants. However, no significant differences in litter N and P contents were observed among the three treatments, possibly due to similar litter species composition across the different restoration treatments [34].

Plant functional groups comprise plant species with similar functional traits and resource utilization, exhibiting comparable responses to the environment and similar effects on major ecosystem processes [35]. The results of this study showed that, across different ecological restoration treatments, the aboveground and underground N contents of the legume functional group were higher than those of the grasses, sedge, and forb functional groups. This is consistent with the biological nitrogen-fixation capability of legumes, whereby symbiotic nodule microorganisms convert atmospheric N_2_ into plant-available N [25]; consistent with this, nodule tissues were observed on the roots of legumes in the MG and SG treatments. However, nitrogen fixation was not quantified, and the higher legume N contents may also partly reflect species identity (which legumes were present) rather than fixation alone. The results also showed that both aboveground and underground P contents of the forb functional group were higher than those of the grasses, sedge, and legume functional groups. This may reflect the deeper, taproot-type root systems of forbs, which could allow access to P in deeper soil layers [36]; however, rooting depth was not measured, and this interpretation remains a hypothesis.

Plant N:P ratios can serve as diagnostic indicators for predicting nutrient limitation and N or P saturation [37]. Koerselman et al. [38] suggested that plants are P-limited when N:P > 16 and N-limited when N:P < 14. Therefore, while nutrient limitation in ecosystem plants cannot be judged solely by a single N:P threshold, it can be inferred that plant communities with relatively lower N:P ratios are more susceptible to N limitation, while those with relatively higher N:P ratios are more susceptible to P limitation [39]. The results of this study showed that overall, the N:P ratios of herbaceous plants, *Salix cupularis* shrubs, and community litter were below 10. The aboveground N:P ratios of herbaceous plants in the SA and SG treatments were significantly lower than those in the MG treatment, which is consistent with the findings of Chai et al. [40]. This suggests that herbaceous plant communities under the SA and SG treatments in the alpine sandy lands of northwestern Sichuan may be more susceptible to N limitation, possibly due to competition for soil nitrogen from *Salix cupularis* shrubs. Regarding plant functional groups, only the aboveground N:P of the forbs functional group and the underground N:P of the legume functional group showed no significant changes among different restoration treatments, while other functional groups exhibited significant differences in response to the restoration treatments. Overall, the forbs functional group showed lower N:P ratios. This indicates significant differences in N and P utilization strategies among different functional groups [34], with forbs demonstrating relatively higher capacity and efficiency in nitrogen utilization.

Overall, plant N and P contents showed significant positive correlations with the Magarlef, Shannon–Wiener, Pielou, and Simpson indices, as well as with plant biomass. This indicates that ecological restoration is conducive to vegetation community recovery while simultaneously enhancing plant capacity for N and P uptake and utilization.

## 4. Materials and Methods

### 4.1. Site Description

**Figure 6:**
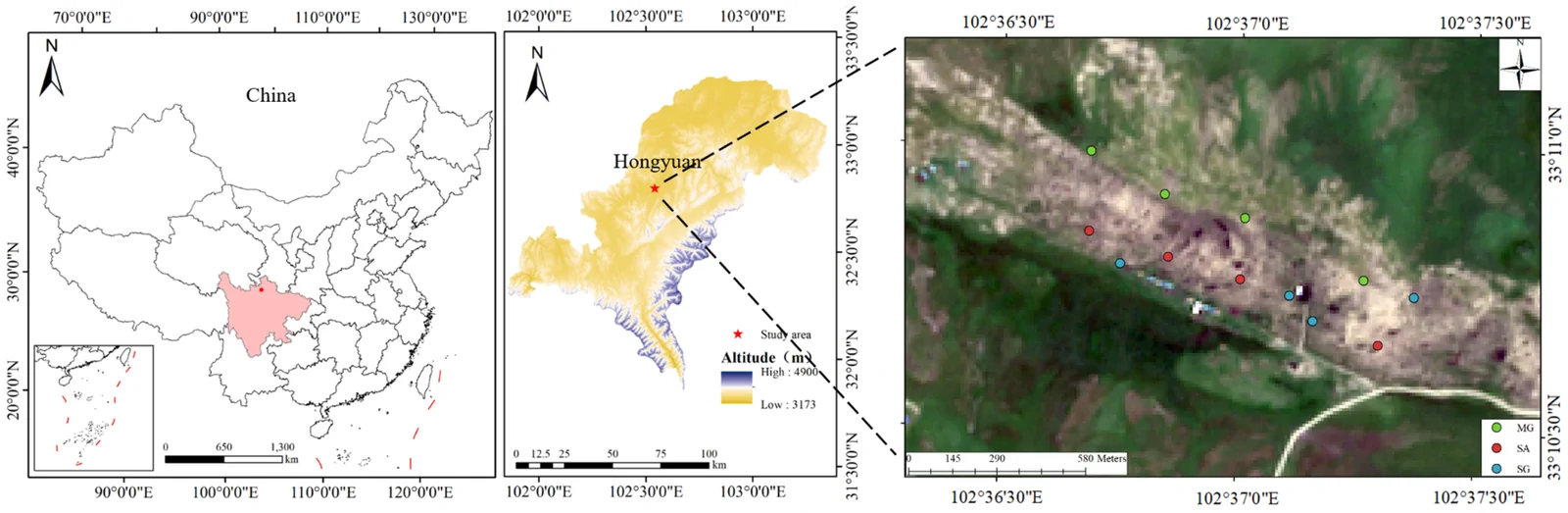
Location of the study area and landscape photos of the sampling plots. Note: MG, planting mixed grasses treatment; SA, planting shrub with *Salix cupularis* alone treatment; SG, planting shrub with *Salix cupularis* plus mixed grasses treatment.

The study area is located in the semi-humid region on the eastern edge of the Qinghai–Tibet Plateau (N 31°51′–33°19′ and E 101°51′–103°23′), the terrain slopes from southeast to northwest, with an altitude ranging from 3210 to 4857 m (Figure 6). The climate is classified as a continental plateau cold temperate monsoon climate, characterized by short springs and autumns, and long winters without summer. The mean annual precipitation is 791.95 mm, mainly concentrated from May to October. The mean annual temperature is 1.1 °C, with the coldest monthly mean temperature of −10.3 °C, the warmest monthly mean temperature of 10.9 °C, and an extreme minimum temperature of −36 °C. The average annual snow cover period is 76 days, and there is absolutely no frost-free period. The area receives ample sunlight with strong solar radiation, featuring an average annual sunshine duration of 2158.7 h and a total annual solar radiation of 6194 MJ·m^−2^. The main soil types in the region include meadow, swamp and peat soils dominate in the region. Due to natural and anthropogenic factors, the alpine grasslands in this region have undergone severe degradation, with desertification occurring in localized areas and showing a trend of gradual expansion [18]. Since the 1980s, restoration efforts in this demonstration area have primarily focused on shrub planting and grass seeding, leading to the formation of three typical ecological restoration treatments with 15 years of continuous restoration: fencing + sand barrier establishment + mixed grass seeding (MG), fencing + sand barrier establishment + shrub planting alone (SA), and fencing + sand barrier establishment + shrub–grass combination (SG). The MG treatment involves managing semi-fixed sandy land through wire fencing and grazing prohibition, establishing plant sand barriers around the perimeter for wind prevention and sand fixation, and artificially replanting perennial grasses. The SA treatment involves fencing + sand barrier establishment combined with planting of *Salix cupularis* cuttings at a spacing of 1–2 m between plants and 2.5 m between rows, without artificial grass seeding. The SG treatment involves fencing + sand barrier establishment combined with planting *Salix cupularis* cuttings and artificially mixed-seeding perennial grasses (Figure 6 and Table 3) [18]. The primary species in SA were *Salix cupularis*, *Lancea tibetica*, and *Leymus secalinus*. The primary species in SG were *Salix cupularis*, *Euphrasia regelii subsp. kangtienensis*, *Anaphalis lacteal*, *Peucedanum praeruptorum*, *Potentilla discolor*, and *Elymus nutans*. The MG treatment was sown with the same perennial grass mixture as SG, so that MG and SG shared an identical sown species composition and differed only in the presence of shrubs.

### 4.2. Experimental Design

In August 2021, at the Highland Sandy Desert Ecological Management Demonstration Base in Hongyuan County, Sichuan Province, three treatments were studied: MG, SA, SG. For each treatment, four replicate restoration areas (*n* = 4) with essentially consistent site conditions (slope and elevation) were selected, and one 25 m × 25 m plot was established in each area. The four replicate areas within each treatment, as well as the areas of different treatments. In each plot of the SA and SG treatments, three *Salix cupularis* shrubs with similar growth conditions were randomly selected; their plant height and crown diameter were measured, and samples of the aboveground parts and underground roots were collected by the cutting and excavation methods, respectively, placed in kraft paper bags, and returned to the laboratory. Within each plot of the MG, SA, and SG treatments, three 1 m × 1 m quadrats were randomly selected (beneath the *Salix cupularis* canopy in SA and SG), in which the species, number, height, and coverage of herbaceous plants were recorded. Surface litter (including litter from herbaceous plants and *Salix cupularis* shrubs) was collected using brushes and mesh bags, and whole herbaceous plants (including aboveground and underground parts) were collected by excavation. Within each plot, the three quadrats and, in SA and SG, the three shrubs were averaged to obtain one plot-level value per variable; the plot was therefore the unit of analysis. The herbaceous plants were classified into four functional groups—grasses, sedges, legumes, and forbs—based on their main plant types and functional characteristics, and their aboveground parts and underground roots were separated.

All plant samples were rinsed clean with tap water, oven-dried at 105 °C for 30 min to deactivate enzymes, then dried at 60 °C to constant weight, and finally sealed in plastic bags for preservation. Biomass of plant functional groups and litter was calculated using the weighing method with cleaned plant samples. The total biomass of the herbaceous plant community was the sum of the biomass of the four plant functional groups.

Total nitrogen content in herbaceous plants, *Salix cupularis*, and litter was determined using H_2_SO_4_-H_2_O_2_ digestion followed by measurement with a Kjeldahl apparatus (Peiou SKD-1000 Fully Automatic Kjeldahl Nitrogen Determination Instrument, Shanghai, China). Total phosphorus content was determined using H_2_SO_4_-H_2_O_2_ digestion followed by the vanadomolybdate yellow colorimetric method (Shimadzu Model 721 spectrophotometer, Shanghai, China, 420 nm wavelength): an amount of 0.1–0.5 g of air-dried soil (accurate to 0.0001 g) was weighed into a 100 mL digestion tube. The sample was moistened with 1 mL of water, then 5 mL of concentrated H_2_SO_4_ was added (analytically pure) and mixed well. H_2_O_2_ (analytically pure) was added in 2 mL portions (repeated as necessary); a small funnel was placed on the tube as a cover, and the sample was digested in a digestion furnace until the solution became colorless or clear; then, heating was continued to drive off the excess H_2_O_2_. After the digest had cooled, it was transferred to a 100 mL volumetric flask, made up to volume, filtered through filter paper, and the filtrate was used for the determination of N and P. A reagent blank was carried out in parallel [41].

### 4.3. Data Statistics and Analysis

The plant community diversity index includes four components: the Margalef richness index (R), the Shannon–Wiener diversity index (H’), the Pielou evenness index (J), and the Simpson dominance index (D). The calculations were performed following the method described in Jiang et al. [18]. The specific formulas are as follows:

Magarlef index:

(1) $$R=\frac{S-1}{\ln N}$$

Shannon–Wiener index:

(2) $$H’=-\sum_{i=1}^{S}P_{i}\ln P_{i}$$

Simpson index:

(3) $$D=1-\sum_{i=1}^{S}{P_{i}}^{2}$$

Pielou index:

*J* = *H’* ∕ ln*S* (4)

where S is the number of plant species occurring in the quadrat; N is the total number of individuals observed; and P_i_ is the relative importance value of the i-th species.

Excel 2022 and Origin 2022 were used for data processing and graphing; SPSS 26.0 was used for statistical analysis. The unit of analysis was the plot (*n* = 4 per treatment). Within each plot, the three 1 m × 1 m quadrats were pooled (biomass and nutrient content averaged) and, in the SA and SG treatments, the three surveyed shrubs were averaged to obtain one plot-level value per variable; quadrats and shrubs were thus treated as subsamples rather than independent replicates. For each response variable, one-way analysis of variance (ANOVA) was used to test differences among the three restoration treatments (MG, SA, SG), with pairwise comparisons by Tukey’s HSD test. Relationships among plant biomass, diversity indices, and N and P contents were evaluated with Spearman’s rank correlation.

## 5. Conclusions

The three restoration treatments established plant communities with substantial total biomass, litter biomass, and diversity; among them, the SG treatment exhibited the highest aboveground biomass and diversity, whereas the MG treatment exhibited the highest underground biomass. The MG and SG treatments exhibited higher grass biomass, while the SA treatment exhibited higher sedge biomass. Across the three treatments, legume biomass accounted for the lowest proportion among plant functional groups. All three restoration treatments exhibited high aboveground N and P contents in herbaceous plants, with the highest values observed in the SG treatment. Compared with the SA treatment, the SG treatment resulted in higher underground N content and N:P ratio in *Salix cupularis* shrubs. Both aboveground and underground N contents of the legume functional group were significantly higher than those of other functional groups, while the forbs functional group exhibited higher aboveground and underground P contents. Under ecological restoration, herbaceous plant N and P contents were significantly positively correlated with plant biomass and diversity. Overall, the SG restoration treatment is more beneficial for enhancing plant community biomass, diversity, and N and P contents in the sandy lands of the semi-humid region on the eastern edge of the Qinghai–Tibet Plateau.

## Figures and Tables

**Table 1:** Effects of ecological restoration treatments on biomass of plant communities.

Treatment	Total Aboveground Biomass (g∙m^−2^)	Total Underground Biomass (g∙m^−2^)	Plant Litter Biomass (g∙m^−2^)
MG	281.99 ± 18.77 c	3840.08 ± 570.96 c	78.61 ± 28.66 b
SA	1773.18 ± 496.68 b	2082.69 ± 430.20 b	358.24 ± 226.62 a
SG	3122.95 ± 351.44 a	3510.06 ± 479.81 a	471.89 ± 229.33 a

Note: MG, planting mixed grasses treatment; SA, planting *Salix cupularis* shrubs alone treatment; SG, planting shrub with *Salix cupularis* plus mixed grasses treatment. Data were presented as means ± SD (*n* = 4). Different lowercase letters indicated significant differences among treatments at *p* < 0.05.

**Table 2:** Effects of ecological restoration treatments on plant diversity index.

Treatment	Margalef	Shannon–Wiener	Pielou	Simpson
MG	2.39 ± 0.13 a	2.12 ± 0.14 a	0.82 ± 0.03 a	0.87 ± 0.02 a
SA	0.74 ± 0.23 b	1.16 ± 0.33 b	0.74 ± 0.08 a	0.62 ± 0.14 b
SG	2.43 ± 0.35 a	2.21 ± 0.07 a	0.82 ± 0.03 a	0.87 ± 0.01 a

Note: MG, planting mixed grasses treatment; SA, planting shrub with *Salix cupularis* alone treatment; SG, planting shrub with *Salix cupularis* plus mixed grasses treatment. Data were presented as means ± SD (*n* = 4). Different lowercase letters indicated significant differences among treatments at *p* < 0.05.

**Table 3:** Basic information on plant communities under different restoration treatments.

Treatment	Elevation (m)	Slop (◦)	Height (m)	Coverage (%)
MG	3416.13 ± 1.68	<5	-	94.3 ± 0.8
SA	3422.03 ± 2.35	<5	1.52 ± 0.11	6.6 ± 1.4
SG	3417.13 ± 3.94	<5	1.64 ± 0.15	81.4 ± 8.9

Note: MG, planting mixed grasses treatment; SA, planting shrub with *Salix cupularis* alone treatment; SG, planting shrub with *Salix cupularis* plus mixed grasses treatment. Data were presented as means ± SD (*n* = 4).

## Data Availability

The original contributions presented in this study are included in the article. Further inquiries can be directed to the corresponding author.
